# Novel Orthopoxvirus and Lethal Disease in Cat, Italy

**DOI:** 10.3201/eid2409.171283

**Published:** 2018-09

**Authors:** Gianvito Lanave, Giulia Dowgier, Nicola Decaro, Francesco Albanese, Elisa Brogi, Antonio Parisi, Michele Losurdo, Antonio Lavazza, Vito Martella, Canio Buonavoglia, Gabriella Elia

**Affiliations:** University of Bari, Valenzano, Italy (G. Lanave, G. Dowgier, N. Decaro, M. Losurdo, V. Martella, C. Buonavoglia, G. Elia);; La Vallonea Veterinary Laboratory, Milan, Italy (F. Albanese);; Centro Veterinario Montarioso, Siena, Italy (E. Brogi);; Istituto Zooprofilattico Sperimentale di Puglia e Basilicata, Putignano, Italy (A. Parisi);; Istituto Zooprofilattico Sperimentale di Lombardia ed Emilia Romagna, Brescia, Italy (A. Lavazza)

**Keywords:** cat, novel orthopoxvirus, orthopoxvirus, viruses, lethal disease, next-generation sequencing, phylogeny, zoonoses, bioterrorism and preparedness, Italy

## Abstract

We report detection and full-genome characterization of a novel orthopoxvirus (OPXV) responsible for a fatal infection in a cat. The virus induced skin lesions histologically characterized by leukocyte infiltration and eosinophilic cytoplasmic inclusions. Different PCR approaches were unable to assign the virus to a defined OPXV species. Large amounts of typical brick-shaped virions, morphologically related to OPXV, were observed by electron microscopy. This OPXV strain (Italy_09/17) was isolated on cell cultures and embryonated eggs. Phylogenetic analysis of 9 concatenated genes showed that this virus was distantly related to cowpox virus, more closely related to to ectromelia virus, and belonged to the same cluster of an OPXV recently isolated from captive macaques in Italy. Extensive epidemiologic surveillance in cats and rodents will assess whether cats are incidental hosts and rodents are the main reservoir of the virus. The zoonotic potential of this novel virus also deserves further investigation.

Orthopoxviruses (OPXVs; family *Poxviridae*, subfamily *Chordopoxvirinae*, genus *Orthopoxvirus*) are complex, double-stranded DNA viruses with ongoing interest because of their potential use as bioterrorism agents and in gene therapy. Variola virus (VARV), the causative agent of smallpox, has been eradicated in nature; however, there is still the possibility of accidental or intentional release, and it is currently classified as a category A biologic agent (*1*). Another concern is the zoonotic potential of some OPXVs, such as monkeypox virus, camelpox virus, buffalopox virus, and cowpox virus (CPXV) (*2**,**3*).

CPXV, which has a wide host range and a distribution restricted to the Eurasian continent, causes localized dermatitis in humans, although severe disease might develop in immunocompromised persons, occasionally with a fatal outcome. Natural hosts for CPXV are wild rodents (*4*), but the infection is acquired mainly through direct contact with cats, which are natural hosts, and rarely by exotic animals and wild species (*5*). Ectromelia virus (ECTV) is the causative agent of mousepox, a severe exanthematous disease of mice in laboratory colonies and has been reported worldwide in several outbreaks and causes high economic losses in biomedical research (*6*). ECTV has never been reported in humans, and little is known regarding its natural distribution and hosts (*7*).

Reports of OPXV infections in animals and humans have largely increased during recent decades, which has enhanced their zoonotic potential and led to the perception of an increasing risk for humans (*8*). For cats, there are several reports of poxvirus infections, but the causative agent has been characterized as CPXV (*9**–**14*) or has not been characterized (*15**–**18*).

We report a case of fatal infection with an OXPV in a household cat. This virus was more closely related to ECTV than to CPXV, putatively representing a novel OPXV species.

## Materials and Methods

### Case Study

**Figure 1:**
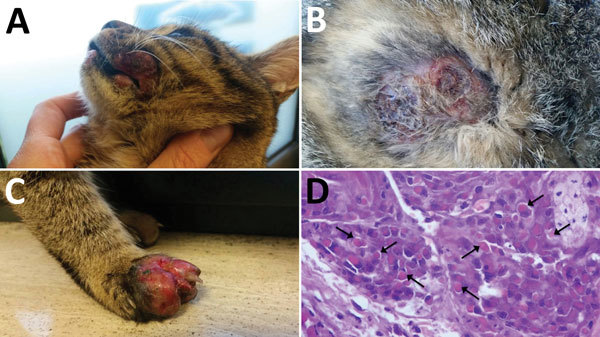
Cat with orthopoxvirus infection, Italy. Ulcerated nodules and plaques were observed on the lips (A), thorax (B), and forelimb (C). Skin punch biopsy specimen (D) showing leukocyte infiltration and cytoplasmic inclusion bodies (arrows) (hematoxylin and eosin stain, original magnification × 60).

A domestic, short-haired, male, 6-month-old cat was brought to a veterinarian because of multicentric, nodular, ulcerative dermatitis (Figure 1). The cat was regularly vaccinated for common feline diseases (feline panleukopenia, rhinhotracheitis, calicivirosis, and chlamydiosis) and showed negative test results for retroviral infections. An antiparasitic product had been applied monthly (Frontline Combo Spot On; Merial, Ingelheim, Germany). The cat was fed a balanced commercial diet and lived indoors, but it had access to outdoors and had a hunting behavior. 

The cat had multiple nodular, plaque-like, ulcerative lesions on its body, particularly on the feet and face. Results of diagnostic testing, including a wood lamp examination, skin scrapings, trichogram, and fungal culture, were negative. Cytological examination showed a mixed inflammatory population of cells with a relevant amount of eosinophils. A blood test showed only a mild leukocytosis with an increase in numbers of lymphocytes, neutrophils, and eosinophils. Because of rapid worsening of its clinical conditions, the cat was euthanized.

### Histopathologic Analysis

We collected multiple skin biopsy specimens for histopathologic analysis by using an 8-mm biopsy punch and fixed these specimens in 10% buffered formalin. Samples were embedded in paraffin, sectioned, and stained with hematoxylin and eosin, according to standard protocols.

### DNA Extraction and PCR Amplification

An OPXV infection was suspected on the basis of clinical presentation and histopathologic analysis. Therefore, we processed histologic preparations for molecular investigations to confirm the presumptive diagnosis. We purified total DNA from a thin section of ≈20 mg of formalin-fixed, paraffin-embedded tissue by using the DNeasy Blood and Tissue Kit (QIAGEN, Hilden, Germany) according to the manufacturer’s instructions.

We tested the DNA extract by using 2 panchordopoxvirus PCRs specific for the variable GC content of the genera included in the subfamily *Chordopoxvirinae* and other unclassified chordopoxviruses (*19*). For the low GC content PCR, we used DNA from a laboratory vaccinia virus Western Reserve strain (VACV-WR) as a positive control. For the high GC content PCR, we used an Orf virus isolated during an outbreak of contagious ecthyma as a positive control. We initially conducted subsequent identification of OPXV by using a PCR specific for the gene coding A-type inclusion protein (*20*), followed by a second PCR specific for the hemagglutinin (HA) gene (*21*). In addition, we performed 2 species-specific PCRs, 1 for ECTV and 1 for CPXV, to further characterize the virus (*22*).

We conducted all PCR amplifications by using an LA PCR Kit (version r.2.1) (Takara Bio, Tokyo, Japan) in a 50-μL reaction containing 1 mmol/L of primers, LA PCR Buffer (Mg^2+^), 8 μL of dNTP mixture (corresponding to 400 mmol/L of each dNTP), 2.5 units of TaKaRa LA Taq polymerase, and 1 μL of template DNA. The cycling protocol used for each assay was programmed as described (*19*). PCR products were subjected to electrophoresis on a 1.5% agarose gel containing a fluorescent nucleic acid marker (GelRed; Bio-Rad Laboratories, Hercules, CA, USA) at 80 V for 45 min and visualized under fluorescent light on the Gel Doc EZ Imaging System with Image Laboratory Software (Bio-Rad Laboratories). PCR products were directly sequenced by Eurofins Genomics GmbH (Ebersberg, Germany). We manually edited and analyzed sequences by using the Geneious platform version 10.1.3 (Biomatters Ltd., Auckland, New Zealand).

### Virus Isolation

After diagnosis of OPXV infection, we collected additional biopsy specimens from skin lesions of the diseased cat intravitam and used them for subsequent virologic investigations. For virus isolation, we used African green monkey kidney fibroblast CV-1 cells and African green monkey kidney epithelial Vero cells. Cells were grown in Dulbecco’s modified minimum essential medium (DMEM) supplemented with 10% fetal bovine serum. Tissues were homogenized in DMEM (10%, wt/vol) and centrifuged at 8,000 × *g* for 10 min. Supernatants were treated with antimicrobial drugs (penicillin 5,000 IU/mL, streptomycin 2,500 μg/mL, and amphotericin B 10 μg/mL) for 30 min, inoculated on partially confluent CV-1 and Vero cell cultures, and incubated at 37°C in a 5% CO_2_ incubator. After an adsorption period of 45 min, DMEM was added. Cells were observed daily for cytopathic effects.

For hematoxylin and eosin staining and indirect immunofluorescence (IIF) assay, we grew cells on coverslips placed in 12-well plates. Cells were mock- or virus-infected and coverslips were harvested at 48 hours postinfection. For detection of inclusion bodies, we fixed cells in Bouin solution for 2 h and stained them with hematoxylin and eosin. For the IIF assay, cells were fixed with 80% acetone for 30 min. Coverslips were rinsed twice with phosphate-buffered saline and incubated 30 min in a humidified chamber at 37°C with a serum sample (diluted 1:50) collected from the ill cat. Coverslips were washed twice with phosphate-buffered saline and incubated with goat anti-cat IgG conjugated with fluorescein isothiocyanate (Sigma-Aldrich, Milan, Italy).

The homogenate of skin biopsy specimens was inoculated onto the chorioallantoic membrane of 12-day-old chick embryos. After 2 days of incubation at 37°C, membranes were collected from the eggs and pock morphology was observed.

### Electron Microscopy

We performed negative staining and electron microscopic analysis of homogenates of skin punch biopsy specimens and supernatants of infected Vero cells that showed an evident cytopathic effect. Samples were frozen and thawed twice and centrifuged at 4,000 × *g* for 20 min and at 9,300 × *g* for 10 min to clarify the supernatant. The second supernatant (82 μL) was then ultracentrifuged in an Airfuge centrifuge (Beckman Coulter, Brea, CA, USA) for 15 min at 21 lbs/in^2^ (82,000 × *g*). The Airfuge was fitted with an A 100 rotor that held six 175-μL test tubes containing specific adapters for 3-mm grids, which enables direct pelleting of virus particles on carbon-coated, formvar copper grids. These grids were stained with 2% sodium phosphotungstate, pH 6.8, for 1.5 min, and observed with a Tecnai G2 Biotwin Transmission Electron Microscope (Field Electron and Ion Company, Hillsboro, OR, USA) operating at 85 kV. We identified poxvirus particles, observed at magnifications of 11,000×–26,500×, on the basis of their typical morphologic characteristics.

### Serologic Analysis

We tested the serum sample collected intravitam from the diseased cat for OPXV antibodies by virus neutralization and IIF assays. We used strains Italy_09/17 isolated from the same cat and VACV-WR in these tests.

For the virus neutralization test, we mixed 2-fold dilutions of heat-inactivated serum (starting at a dilution of 1:2) with 100 50% tissue culture infective doses of virus in 96-well microtiter plates. After incubation at room temperature for 60 min, 2 × 10^4^ CV-1 cells were added to each well. Plates were read after 4 days of incubation at 37°C in a humidified atmosphere of 5% CO_2_. 

For the IIF assay, we fixed confluent monolayers of CV-1 cells grown on coverslips and infected with strain Italy_09/17 or VACV-WR with 80% acetone. We tested 2-fold dilutions of heat-inactivated serum (diluted 1:20 to 1:5,120) by using 1 coverslip/dilution. Goat anti-cat IgG conjugated with fluorescein isothiocyanate was used as a secondary antibody (Sigma-Aldrich).

### Next-Generation Sequencing

For DNA extraction, we obtained virus stocks from semipurified virus particles. In brief, we infected CV-1 cells with strain Italy_09/17. At 48 hours postinfection, the cell medium was collected and nuclei and cell debris were discarded by centrifugation at 1,000 × *g* for 10 min at 4°C. We extracted virus DNA by using a QIAamp Cador Pathogen Mini Kit (QIAGEN) according to the manufacturer’s instructions.

We quantified DNA by using the Fluorometric Qubit dsDNA High Sensitivity Assay Kit (Thermo Fisher Scientific, Waltham, MA, USA). We prepared a genomic DNA library by using the Nextera DNA Sample Prep Kit (Illumina, San Diego, CA, USA) according to the manufacturer’s protocol and performed a size-selection step manually by using Ampure XP magnetic beads (Beckman Coulter). We performed quality control analysis of the sample library by using the QIAxcel Advanced System with QIAxcel ScreenGel Software 1.4.0 (QIAGEN). We normalized library samples as suggested by QIAGEN and performed sequencing by using a MiSeq instrument, version 2, and a MiSeq Reagent Kit (Illumina).

### Genome Annotation and Comparison

We obtained 1,497,762 paired reads in next-generation sequencing (NGS) experiments (Illumina); these reads had an average length of 155.4 bp. We performed quality control of reads by using FastQC (*23*) and sequence trimming by using the plugin Trim Ends in Geneious software version 10.1.3 (https://www.geneious.com/). We filtered NGS sequences by using the genome of African green monkey (*Chlorocebus sabeus*), which yielded 217,236 unmapped reads. We used these unmapped reads for de novo assembling of the feline OPXV genome by using the Geneious Assembler.

We annotated the nearly full-length genome sequence of the Italy_09/17 isolate by using ECTV strain Naval as reference (GenBank accession no. KJ563295). We performed genome annotation by using FindORFs software in Geneious version 10.1.3 and a set of reference sequences, including ECTV Naval (accession no. KJ563295), ECTV Mos (accession no. AF012825), CPXV BR (accession no. AF482758), and VACV COP (accession no. 35027) for comparison. We further analyzed open reading frames that remained unassigned or with a lower similarity to the reference sequences by using MyOrfeome (http://myorfeome.sourceforge.net).

### Phylogenetic Analysis

For characterization of the OPXV strain, we used the strategy proposed by Emerson et al. (*24*). We selected 9 coding sequences from the genome of the feline OPXV strain Italy_09/17: A7L, early transcription factor/VETF large subunit; A10L, major core protein; A24R, RNA polymerase 132; D1R, messenger RNA capping enzyme, large subunit; D5R, DNA-independent NTPase (DNA replication); E6R, hypothetical protein; E9L, DNA polymerase; H4L, RNA polymerase-associated protein; and J6R, RNA polymerase 147. Gene designations refer to the VACV COP genome. We aligned concatenated genome sequences of OPXVs representative of North American and Old World (African and Eurasian) viruses by using Geneious version 10.1.3 and the MAFFT algorithm (*25*). After searching the GenBank database, we retrieved complete HA gene sequences of 2 feline-derived human OPXV strains (accession nos. EF612709 and FJ445747) and of an OPXV isolated from captive macaques (accession no. KY100116) and aligned them with cognate OPXV sequences.

We performed phylogenetic analysis for concatenated DNA alignments with Bayesian inference by using 4 chains run for >1 million generations (*26**,**27*). We used ModelTest software (http://evomics.org/resources/software/molecular-evolution-software/modeltest/) to identify the most appropriate model of evolution for the entire dataset and for each gene individually. The identified program settings for all partitions, under the Akaike Information Criteria, included 6 character states (general time reversible model), a proportion of invariable sites, and a gamma distribution of rate variation across sites. We deposited nucleotide sequences of strain Italy_09/17 used for phylogeny in GenBank (accession nos. MF578930–9).

### Detection of Other Pathogens

We subjected nucleic acids extracted from freshly collected skin biopsy specimens and serum of the affected cat to a TaqMan assay for detection of canine parvovirus 2/feline panleukopenia virus (*28*) and to a minor groove binder probe assay for rapid discrimination between true feline panleukopenia virus strains and antigenic variants of canine parvovirus 2 (*29*). We also used DNA extracts to detect proviral DNA of feline immunodeficiency virus (*30*) and feline leukemia virus (*31*) and DNA of feline hemoplasmas (*32*) and feline herpesvirus (*33*). We screened RNA extracts by real-time PCR or conventional reverse transcription PCR specific for carnivore coronaviruses (*34*) and caliciviruses (*33**,**35*).

## Results

### Histopathologic Analysis

**Figure 2:**
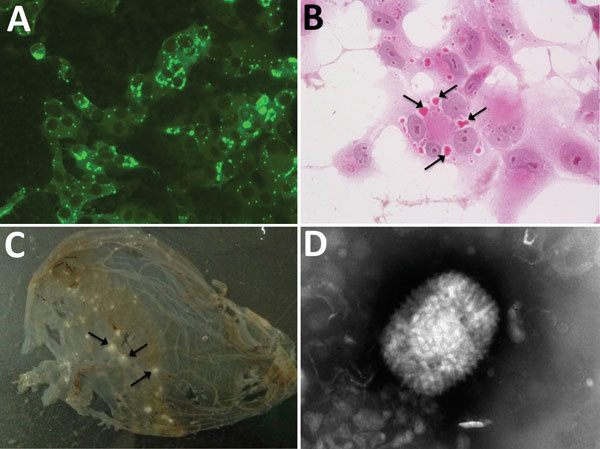
Analysis of an orthopoxvirus isolated from an infected cat, Italy. A) Cytoplasmic fluorescence in infected Vero cells using serum from the diseased cat (original magnification ×400). B) Cytoplasmic inclusion bodies (arrows) in infected Vero cells (hematoxylin and eosin stain, original magnification ×400). C) Pocks (arrows) in the inoculated chorioallantoic membrane of a 12-day-old chick embryo. D) Electron micrograph of orthopoxvirus-like particle from infected Vero cells. The virus preparation was negative-stained with sodium phosphotungstate (original magnification ×25,000).

Histopathologic analysis of multiple skin specimens showed mild hyperplasia of the epidermis and the follicular wall. Many roundish to oval brightly eosinophilic inclusion bodies were clearly evident in the cytoplasm of both epidermal and follicular keratinocytes, including in a few sebocytes. The morphology of cells suggested a possible OPXV infection (Figure 2, panel A). Nodular to diffuse dermatitis caused by mixed inflammatory cells was also present in dermis and hypodermis; those cells were mainly represented by eosinophils, histiocytes, and lymphocytes, together with few plasma cells and neutrophils.

### Molecular Investigations

Molecular analysis of formalin-fixed, paraffin-embedded tissues showed positive results for the low GC panchordopoxvirus PCR and negative results for the high GC panchordopoxvirus PCR (*19*). This pattern of amplification was consistent with an OPXV infection. Amplification of the gene coding for the A-type inclusion protein generated an amplicon of ≈1,237 bp, and amplification of the HA gene (*20**,**21*) generated an amplicon of 846 bp, which are expected sizes for these genes in ECTV. All samples collected from the cat were negative for other pathogens by the molecular assays used.

Sequence analysis of the HA gene of strain Italy_09/17 showed high nucleotide identity (98%) with that of CPXV strain Germany (GenBank accession no. HQ420897) and to feline-derived human poxvirus IT1 (accession no. EF612709). In addition, strain Italy_09/17 was also highly related to most of the ECTV strains in GenBank; the highest (97%) nucleotide identity was with ECTV strain Naval (accession no. KJ563295). Strain Italy_09/17 showed positive results in the ECTV-specific PCR and negative results in the CPXV-specific PCR (*22*) (Table 1).

**Table 1:** PCR approach for identification of viruses of the subfamily Chordopoxvirinae*

Specificity	Target gene	Reference	Result	Amplicon, bp	Sequence	First match by BLAST analysis†	Nucleotide identity, %
Panchordopoxvirus, low GC	Insulin metalloproteinase-like protein gene/IMV membrane protein gene	(*19*)	+	220	+	ECTV Naval KJ563295	100
Panchordopoxvirus, high GC	Insulin metalloproteinase-like protein gene/IMV membrane protein gene	(*19*)	ND	ND	ND	ND	ND
Eurasian/African OPXVs	A-type inclusion protein gene	(*20*)	+	1, 237‡	+	CPXV Germany 91–3 DQ437593	98
Eurasian/African OPXVs	Hemagglutinin gene	(*21*)	+	864§	+	Feline poxvirus ITA2 FJ445747	96
ECTV	Hemagglutinin gene	(*22*)	+	150	+	ECTV Naval KJ563295	99
CPXV	Hemagglutinin gene	(*22*)	ND	629–677	ND	ND	ND

*CPXV, cowpox virus: ECTV, ectromelia virus; IMV, intracellular mature virus; ND, not determined; OPXV, orthopoxvirus; +, sequence obtained. †https://blast.ncbi.nlm.nih.gov/Blast.cgi?PAGE_TYPE=BlastSearch. GenBank accession numbers are provided. ‡Expected size for CPXV: 1,601 or 1,673 bp; expected size for ECTV: 1,220 bp. §Expected size for CPXV: 942 bp; expected size for ECTV: 846 bp.

### Virus Isolation

Virus isolation from freshly collected skin biopsy specimens was successful with Vero and CV-1 cells. We observed a cytopathic effect at 48 hours postinfection that showed rounding of cells, increased granularity, and detachment from the monolayer. In Vero cells, a cytopathic effect was less evident than in CV-1 cells. Cells stained with hematoxylin and eosin contained large eosinophilic cytoplasmic inclusion bodies that were compatible with infection by poxviruses, including CPXV (*36*) and ECTV (*37*) (Figure 2, panel B).

The IIF assay showed granular fluorescence areas that displayed the morphology of the inclusion bodies in cell cytoplasms (Figure 2, panel C). Both CV-1 and Vero cells showed positive IIF assay results, but there was no fluorescence staining in the negative control.

At 48 hours postinoculation on embryonated eggs, virus produced superficial pocks on the chorioallantoic membrane. Most of these pocks were small (diameter 1.0 mm), gray, and had central hemorrhages. Few (3%–5%) pocks were larger (1.8 mm in diameter), white, and without hemorrhages.

### Electron Microscopy

Many typical brick-shaped virions (≈320 × 240 nm) morphologically related to the genus *Orthopoxvirus* were observed by negative staining and electron microscopy. We observed these results for skin punch biopsy specimens and cell culture supernatants.

As in a previous study (*15*), few particles showed the characteristic ribbon structure of the M form of vaccinia virus (*38*) (Figure 2, panel D), which is usually prevalent in fresh preparations collected during acute-phase infections. Most virions were slightly larger, showed a uniform electron density, and had a thick capsule outlined by a ragged edge (i.e., the morphologic aspect known as the C form), which are less infective and prevalent during evolution of a chronic infection.

### Serologic Analysis

The infected cat was negative by virus neutralization for strain Italy_09/17 and reference VACV isolates. However, the IIF assay detected antibody titers of 1:1,280 for virus Italy_09/17 and 1:640 for VACV-WR.

### Identification of a Novel OPXV by NGS

We used 217,236 paired reads for de novo assembling and obtained 3 contigs (contig one, 195,015 bp; contig two, 21,014 bp; and contig three, 1,596 bp) and a quality score >99%. The mean coverage of the assembled contigs was 61×. The 9 open reading frames (A7L, A10L, A24R, D1R, D5R, E6R, E9L, H4L, and J6R) used for OPXV characterization were mapped in contig 1, and their sequences (total 27,228 nt) were concatenated and aligned with concatenated cognate sequences of selected OPXVs. In addition, because the HA gene of 2 feline-derived human virus isolates was available in the sequence databases, we performed an alignment based on the HA gene. We conducted phylogenetic analysis on the basis of the 9 concatenated sequences by using Bayesian inference. Posterior probabilities percentages were consistently high (>90%) for all clades on phylograms, which supported inferred phylogenetic relationships.

**Figure 3:**
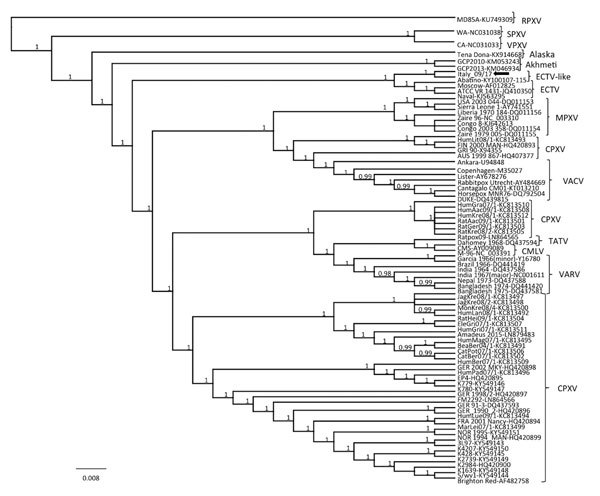
Phylogenetic relationship of extant orthopoxviruses with a feline poxvirus isolated from a cat, Italy. Phylogenetic tree shows 27,228 nt concatenated alignment of 9 coding gene (A7L, A10L, A24R, D1R, D5R, E6R, E9L, H4L, and J6R) sequences of orthopoxvirus. Gene designations refer to the VACV-COP genome (GenBank accession no. M35027). Posterior output of the tree was derived from Bayesian inference using 4 chains run for >1 million generations, a general time-reversible model, a proportion of invariable sites, a gamma distribution of rate variation across sites, and a subsampling frequency of 1,000. Posterior probability values >0.95 are indicated on the tree nodes. The black arrow indicates the feline poxvirus Italy_09/17 isolated in this study. Raccoonpox virus strain MD85A was used as an outgroup. Strain name, host and year of detection, location of origin, and GenBank accession numbers for orthopoxviruses used for phylogeny are shown in Table 2). Scale bar indicates nucleotide substitutions per site. CMLV, camelpox virus; CPXV, cowpox virus; ECTV, ectromelia virus; MPXV, monkeypox virus; RPXV, raccoonpox virus; SPXV, skunkpox virus; TATV, taterapox virus; VACV, vaccinia virus; VARV, variola virus; VPXV, volepox virus.

In the consensus phylogenetic tree (Figure 3), we found that strain Italy_09/17 was distantly related to other OPXVs, including all 10 CPXV lineages (*39*) and other recently identified, deep-branching OPXVs (*40**,**41*) and displayed a closer relatedness with ECTV prototypes, albeit forming a separate cluster. This cluster also included strain Abatino, which was recently isolated from a poxvirus outbreak in a captive colony of Tonkean macaques in Italy (*42*). Nucleotide identity of strain Italy_09/17 with strain Abatino was 99.66% and identity with reference ECTVs was 98.11%–98.13%. Higher nucleotide identities were found among ECTVs (99.97%–99.99%) and between VARV-Garcia1966 (variola minor) and VARV-India1967 (variola major) (99.68%). Thus, on the basis of current OPXV criteria of species demarcation, the cat and macaque isolates should be considered prototypes of a novel OPXV. Also, for the HA gene, strain Italy_09/17 appeared more closely related to strain Abatino (99.79% nt identity) than to feline-derived human OPXV strains (95.83%–95.99% nt identity) that were identified in Italy in 2009, for which a full-length genome and concatenated genes used for species demarcation using phylogeny are not available (*43*).

**Table 2:** Viruses (n = 81) used for phylogenetic analysis of an orthopoxvirus isolated from a cat, Italy*

Species	Strain	Host	Year	Location of origin	GenBank accession nos.
Akhmeti	GCP2010	Human	2010	Georgia	KM053243–51
Akhmeti	GCP2013	Human	2013	Georgia	KM046934–42
Alaska	Tena Dona	Human	2015	Alaska, USA	KX914668–76
CPXV	3L97	Cat	1977	UK	KY549143
CPXV	AUS 1999 867	Cat	1999	Texing, Austria	HQ407377
CPXV	GRI 90	Human	1990	Moscow, Russia	X94355
CPXV	FIN 2000 MAN	Human	2000	Tohmajärvi, Finland	HQ420893
CPXV	HumLit08/1	Human	2008	Vilnius, Lithuania	KC813493
CPXV	HumAac09/1	Human	2009	Aachen, Germany	KC813508
CPXV	RatAac09/1	Rat	2009	Aachen, Germany	KC813501
CPXV	RatGer09/1	Rat	2009	Germering, Germany	KC813503
CPXV	HumKre08/1	Human	2008	Krefeld, Germany	KC813512
CPXV	RatKre08/2	Rat	2008	Krefeld, Germany	KC813505
CPXV	Ratpox09	Rat	2009	Marl, Germany	LN864565
CPXV	HumGra07/1	Human	2007	Graz, Austria	KC813510
CPXV	GER 1998/2	Human	1998	Eckental, Germany	HQ420897
CPXV	GER 91–3	Human	1991	Munich, Germany	DQ437593
CPXV	FM2292	Common vole	2011	Baden-Wuerttemberg, Germany	LN864566
CPXV	MarLei07/1	Mara	2007	Leipzig, Germany	KC813499
CPXV	HumLue09/1	Human	2009	Lübeck, Germany	KC813494
CPXV	GER_1990_2	Human	1990	Bonn, Germany	HQ420896
CPXV	FRA 2001 Nancy	Human	2001	Nancy, France	HQ420894
CPXV	NOR 1995	Cat	1994	Bergen, Norway	KY549151
CPXV	NOR 1994_MAN	Human	1994	Bergen, Norway	HQ420899
CPXV	K1639	Cat	2000	Bristol, UK	KY549148
CPXV	K2739	Cat	2000	Bristol, UK	KY549149
CPXV	5/wv1	Cheetah	1972	London, UK	KY549144
CPXV	K4207	Cat	2000	Bristol, UK	KY549150
CPXV	Brighton Red	Human	1937	Brighton, UK	AF482758
CPXV	K2984	Cat	2000	Bristol, UK	HQ420900
CPXV	K428	Cat	2000	Bristol, UK	KY549145
CPXV	K779	Cat	2000	Bristol, UK	KY549146
CPXV	K780	Cat	2000	Bristol, UK	KY549147
CPXV	EP4	Elephant	1980	Hameln, Germany	HQ420895
CPXV	HumPad07/1	Human	2007	Paderborn, Germany	KC813496
CPXV	GER 2002 MKY	Marmoset	2002	Göttingen, Germany	HQ420898
CPXV	BeaBer04/1	Beaver	2004	Berlin, Germany	KC813491
CPXV	CatBer07/1	Cat	2007	Berlin, Germany	KC813502
CPXV	HumBer07/1	Human	2007	Berlin, Germany	KC813509
CPXV	HumMag07/1	Human	2007	Magdeburg, Germany	KC813495
CPXV	CatPot07/1	Cat	2007	Potsdam, Germany	KC813506
CPXV	Amadeus 2015	Horse	2015	Germany	LN879483
CPXV	EleGri07/1 D	Elephant	2007	Grimmen, Germany	KC813507
CPXV	HumGri07/1 D	Human	2007	Grimmen, Germany	KC813511
CPXV	RatHei09/1	Rat	2009	Heidelberg, Germany	KC813504
CPXV	JagKre08/1	Jaguarundi	2008	Krefeld, Germany	KC813497
CPXV	JagKre08/2	Jaguarundi	2008	Krefeld, Germany	KC813498
CPXV	MonKre08/4	Mongoose	2008	Krefeld, Germany	KC813500
CPXV	HumLan08/1	Human	2008	Landau, Germany	KC813492
CMLV	M-96	Camel	1996	Kazakhstan	NC003391
CMLV	CMS	Camel	1970	Iran	AY009089
ECTV	Naval	Mouse	1996	USA	KJ563295
ECTV	Moscow	Mouse	1947	Moscow, Russia	NC004105
ECTV	ATCC VR 1431	Human	1987	China	JQ410350
ECTV-like	Abatino	Primate	2015	Italy	KY100107–15
ECTV-like	Italy_09/17	Cat	2017	Italy	MF578930–8
MPXV	Liberia 1970 184	Human	1970	Liberia	DQ011156
MPXV	Zaire 96	Human	1996	Zaire, Democratic Republic of the Congo	NC003310
MPXV	Sierra Leone 1	Human	2004	Sierra Leone	AY741551
MPXV	Congo 8	Human	1970	Zaire, Democratic Republic of the Congo	KJ642613
MPXV	Congo 2003 358	Human	2003	Democratic Republic of the Congo	DQ011154
MPXV	USA 2003 044	Prairie dog	2003	USA	DQ011153
MPXV	Zaire 1979 005	Human	1979	Zaire, Democratic Republic of the Congo	DQ011155
RPXV	MD85A	Raccoon	1964	Maryland, USA	KU749309
SPXV	WA	Skunk	1978	Washington, USA	NC031038
TATV	Dahomey 1968	Gerbil	1968	Dahomey, Benin	NC008291
VACV	Rabbitpox Utrecht	Rabbit	1941	Utrecht, the Netherlands	AY484669
VACV	Horsepox MNR76	Horse	1976	Mongolia	DQ792504
VACV	Copenhagen	Human	1990	Copenhagen, Denmark	M35027
VACV	Cantagalo CM01	Dairy cow	1999	Brazil	KT013210
VACV	Ankara	Human	1998	Austria	U94848
VACV	DUKE	Human	1970	USA	DQ439815
VACV	Lister	Human	2004	Tokyo, Japan	AY678276
VARV	Bangladesh 1974	Human	1976	Bangladesh	DQ441420
VARV	Brazil 1966	Human	1966	Brazil	DQ441419
VARV	India 1967 (major)	Human	1967	India	NC001611
VARV	Garcia 1966 (minor)	Human	1966	Russia	Y16780
VARV	Bangladesh 1975	Human	1975	Bangladesh	DQ437581
VARV	India 1964	Human	1964	India	DQ437586
VARV	Nepal 1973	Human	1973	Nepal	DQ437588
VPXV	CA	Common vole	1985	California, USA	NC031033

*CMLV, camelpox virus; CPXV, cowpox virus; ECTV, ectromelia virus; MPXV, monkeypox virus; RPXV, raccoonpox virus; SPXV, Skunkpox virus; TATV, taterapox virus; VACV, vaccinia virus; VARV, variola virus; VPXV, volepox virus.

## Discussion

OPXV infection in cats is frequently observed, and OPXV transmission from cats to humans has been demonstrated or at least suspected on several occasions (*8**,**11**,**14*,*43**–**47*). Cats are susceptible to CPXV infection, for which they represent only incidental hosts, as are humans, cattle, horses, and dogs. The virus is usually transmitted to cats by hunted rodents; cat-to-cat transmission is apparently rare (*47*). In contrast, ECTV has a host range restricted to laboratory mice, and cat or human infections have not been reported (*6*).

Additional OPXVs, such as raccoonpox virus and skunkpox virus, have been reported in wildlife and infect carnivores (*48*). Conversely, cats have been found to be susceptible to members of the genus *Parapoxvirus*, raccoonpox virus, and uncharacterized poxviruses (*49*). There are >400 reports of OPXV infection in domestic cats, but the total number of feline cases is considered to be much greater (*5*). Despite this large number of reports, genetic characterization of the detected poxvirus has been achieved in only a few instances. Thus, circulation in cats of other OPXVs cannot be ruled out.

We report detection of an OPXV strain that caused a fatal infection in a cat. The virus was not a classical CPXV, which is common in felids. Analysis of 9 concatenated genes showed that the poxvirus detected was only distantly related to all CPXV lineages currently known and formed a separate cluster with respect to ECTV, with which it was strictly related and grouped with an OPXV strain recently isolated from captive macaques in Italy (*42*). These 2 viruses had lower genetic identity with ECTV than that observed with reference ECTVs and between variola minor virus and variola major virus. Therefore, these ECTV-like poxviruses likely represent a novel OPXV species. However, the true animal reservoir of this novel OPXV needs to be assessed, and the idea that wild rodents can act as carriers for the new virus cannot be ruled out.

If one considers the close relatedness between strain Italy_09/17 and ECTV, which has been detected only in laboratory animals, it could be speculated that an ECTV-like virus circulating in wild rodents has resulted in ECTV strains adapted to laboratory mice. Alternatively, an ECTV strain might have escaped from laboratory mice and adapted to wild conditions. In addition, the zoonotic potential of the feline ECTV-like OPXV deserves an in-depth investigation. Feline poxvirus was also related to an unclassified OPXV, which was detected in a human in Italy almost 10 years ago and for which only partial HA gene has been identified (*43*). Consequently, this feline poxvirus could represent a threat to human health. Thus, veterinarians and cat breeders and owners should be aware of this additional risk associated with handling of cats with skin lesions.
